# Algal Biomass Analysis by Laser-Based Analytical Techniques—A Review

**DOI:** 10.3390/s140917725

**Published:** 2014-09-23

**Authors:** Pavel Pořízka, Petra Prochazková, David Prochazka, Lucia Sládková, Jan Novotný, Michal Petrilak, Michal Brada, Ota Samek, Zdeněk Pilát, Pavel Zemánek, Vojtěch Adam, René Kizek, Karel Novotný, Jozef Kaiser

**Affiliations:** 1 Institute of Physical Engineering, Faculty of Mechanical Engineering, Brno University of Technology, Technická 2896/2, Brno 61669, Czech Republic; E-Mails: pavel.porizka@ceitec.vutbr.cz (P.P.); david.prochazka@ceitec.vutbr.cz (D.P.); jan.novotny@ceitec.vutbr.cz (J.N.); 2 BAM–Federal Institute for Material Research and Testing, Richard Willstätter-Straβe 11, Berlin D-12489, Germany; 3 CEITEC—Central European Institute of Technology, Brno University of Technology, Technická 3058/10, Brno 61600, Czech Republic; E-Mails: pekucero@seznam.cz (P.P.); lucia.sladkova@ceitec.vutbr.cz (L.S.); michal.petrilak@ceitec.vutbr.cz (M.P.); michal.brada@ceitec.vutbr.cz (M.B.); vojtech.adam@mendelu.cz (V.A.); kizek@sci.muni.cz (R.K.); codl@sci.muni.cz (K.N.); 4 Institute of Scientific Instruments of the Academy of Science of the Czech republic, v.v.i., Královopolská 147, Brno 612 64, Czech Republic; E-Mails: osamek@isibrno.cz (O.S.); pilat@isibrno.cz (Z.P.); zemanek@isibrno.cz (P.Z.); 5 Department of Chemistry, Faculty of Science, Masaryk University, Kotlářská 2, Brno 61137, Czech Republic

**Keywords:** Laser-Induced Breakdown Spectroscopy, LIBS, Laser-Ablation Inductively Coupled Plasma coupled with Mass Spectroscopy and Optical Emission Spectroscopy, LA-ICP-MS, LA-ICP-OES, ICP-OES, Raman spectroscopy, algae, algal biomass, biofuel, bioremediation

## Abstract

Algal biomass that is represented mainly by commercially grown algal strains has recently found many potential applications in various fields of interest. Its utilization has been found advantageous in the fields of bioremediation, biofuel production and the food industry. This paper reviews recent developments in the analysis of algal biomass with the main focus on the Laser-Induced Breakdown Spectroscopy, Raman spectroscopy, and partly Laser-Ablation Inductively Coupled Plasma techniques. The advantages of the selected laser-based analytical techniques are revealed and their fields of use are discussed in detail.

## 1. Introduction

Expanding economies are still technologically dependent on crude oil, while the decreasing amount of oil deposits moves up prices of crude oil and related gasoline [1]. Trends are observed in searching for alternatives to fossil fuels. Another challenge lies in satisfying global energy needs in a way that would decrease the level of environmental pollution. This challenge can be faced with renewable energy sources [2]. Algae are a possible energy source which could solve both issues. Amongst prospective alternatives to fossil fuel, algae have become one of the most significant without competing for arable land [3–5]. Algae convert solar energy into lipids, carbohydrates and proteins via photosynthesis and then further processing of these primary metabolites can take place. Algae have a per-acre per-year yield that is 200 times higher than the best-performing plant/vegetable oils [6], while some algal strains are also capable of doubling their mass several times per day [7].

The complex study of algae as a future biodiesel and biomass feedstock was initiated by Williams and Laurens [3]. They summarized the collection of oil-producing microalgae. Great emphasis was placed on understanding the biochemistry of the algal strains and on the development of algal production systems. They pointed out the influence of the biochemical composition of the biomass (*i.e.*, the lipid content) on the economics of the biofuel production. They concluded that algal biofuels are potential valuable alternative to “traditional” biofuels. However, the non-profitable economics (considering the oil prices in the 1990s) of the biofuel production caused the termination of the long-term research program funded by the U.S. Department of Energy [4]. The possible replacement of fossil fuels by algal biofuels is a matter of future research and commercialization of the production process [8,9]. Algae may provide an effective solution, but several challenging aspects need to be overcome [3,10,11], e.g., light use efficiency, high amount of oil production in the algal cell, daily crop harvest, effective algal biomass to 3rd generation biofuel conversion, and the improvement of the entire system economics [3].

Algae can be grown in open pond systems [6,12–14] and bioreactors [15] with a possibility of a daily harvest. Algal ponds can as well serve for waste water treatment [13,14], which should result in the cost reduction. Every algal strain has to be grown under optimized conditions to obtain high amounts of crop harvest per day, e.g., sufficient sunlight, nutrients, and protection against natural pests. Individual algal strains have different properties and react differently to the conditions in which they grow. On the other hand, algal strains can easily adapt to their new environment [3]. Bioremediation is the ability of the algal strain to grow in polluted water and even prosper to increase its yielded lipid content [16–18]. Algae can also be used as bio-indicators of water pollution level, e.g., to determine the presence of heavy metals [19,20]. In order to reduce expenses for algae cultivation, it is possible to use various sources for nutrient control, including the agricultural waste waters [5]. Environmental and climate changes can be traced in coralline algae–in mineralized algae species that are an excellent record of this information [21].

It is noteworthy that algae may be also used in other fields with potential economic impact [22,23], such as the food, cosmetic and pharmaceutical industries. A review covering majority of literature sources concerning marine algae products was done by Blunt *et al.* [24]. One of the studies is focused on the possible processing of an algal cell in order to yield more products at once [25]. Cardozo *et al.* [22] report on the importance of algae in the food industry in numerous countries, where the emphasis has moved from wild harvests to farming and controlled cultivation in order to produce valuable products on a large scale. The investigation of the algal chemical composition and related products are nowadays promising research areas in the pharmaceutical industry. Algal products may be used in cancer [26] and HIV [27] treatment. In civil engineering, flat panel airlift reactors for lipid production by the algal strain *Chlorella vulgaris* were already installed [28]. The reactor was designed as the renewable energy stock based on the algal biomass production. Another step in this research and development is the construction of a building with a bioreactor facade in Hamburg, Germany [29], where the energy is supplied by the growth of the algal biomass.

Despite the main aim of this article we briefly review the analysis of algae utilizing other techniques. Light Detection and Ranging (LIDAR) is an optical remote sensing technique that may be used for the analysis of larger areas [30]. Phytoplankton in the delta of the river Po was monitored utilizing fluorescence LIDAR systems placed on a van [31], oceanographic ship and airplane [32]. Laser-induced fluorescence has a long history of applications in the detection of marine algae; in the year 1972 a series of measurements was made employing a dye laser, ruggedized for airborne use [33].

Algae are mainly composed of carbohydrates, proteins, nucleic acids, and lipids, where carbohydrates and lipids are responsible for the energy storage [3]. It was reported that the lipid content in the lipid bodies depends on the growth and the nutrient status of algae. Various techniques are employed for obtaining elemental or molecular information of an algal strain. For instance, nuclear magnetic resonance (NMR) spectroscopy was applied to the analysis of plants, fungi and algae by Martin [34]. A non-invasive *in vivo* measurement employing NMR spectroscopy revealed details of the nitrogen and carbon metabolism in real time [35,36]. NMR spectroscopy was utilized to give characteristic fingerprints of the lipid extractions from algal samples, while marine algal strains and samples from the Lagoon of Venice were compared [37]. Danielewicz *et al.* [38] studied the intact triacylglycerol composition of four microalgae species using MALDI-TOF-MS (matrix-assisted laser desorption and ionization time-of-flight mass spectrometry) and ^1^H-NMR spectroscopy. Moreover, MALDI-TOF-MS was employed in other studies for comparison of various algal strains [39–41].

The fast estimation of the algae lipid content is possible by employing Raman spectroscopy. Raman spectra—in the sense of a fingerprint—give information about the saturated and unsaturated fatty acids in the lipid body [42–45]. Samek *et al.* [42] showed that it is feasible to calculate the iodine value (IV) from Raman spectra. IV quantifies the degree of unsaturation and is mainly used in the biodiesel industry [46]. Moreover, the analysis of fatty acid composition in algae by gas chromatography—mass spectrometry (GC-MS) is also possible; however, it is a time-consuming technique [47,48].

Atomic spectroscopy techniques are in general the most commonly used for elemental analysis [49]. Inductively Coupled Plasma techniques, among others, have been the most commonly used technique in any field of interest. It was shown that Laser-Ablation Inductively Coupled Plasma Mass Spectrometry (LA-ICP-MS) is an appropriate method for detection of environmental changes over the year (or even decades) in coralline algae [50–55]. This method is characterized by high spatial-resolution that is required to detect long-lived coralline algae, because seasonal growth increment widths range approximately from 230 to 330 μm/yr [50].

Winefordner *et al.* [49] proposed another laser-based technique, Laser-Induced Breakdown Spectroscopy (LIBS), as a future superstar for elemental analysis of various samples in any state of matter. LIBS has the added advantage that the analysis can be performed remotely, provided optical access can be established between the instrument and the target. When analyzing a sample with other techniques the sample has to be presented to the instrument. This of course is not the case of LIBS, because all interactions between the system and the target can be purely optical. Therefore, LIBS has an extremely competitive position and excels if remote, *in-situ*, real time analysis is required. For instance, in analyzing a water jet containing algal suspension where only optical access using either an optical fiber or a telescope can be used. The most appropriate applications are thus found to be those which prefer remote quantitative or qualitative analysis, without any physical contact with the sample.

Moreover, the sample does not have to be prepared for analysis using solvents and any surface contaminants can be ablated off the sample before carrying out a measurement. This makes LIBS ideal for algal strain analysis where the target may be in the form of dried biomass as algal suspension. Specially engineered systems can be designed and assembled for each analytical problem allowing fast decisions to be made concerning the identification/analysis of target materials which can then be immediately analyzed, sorted and labelled. In this review, LIBS is critically evaluated and considered as a mature technique capable of competing with other techniques for elemental analysis.

Furthermore, the combination of Raman spectroscopy (chemical composition) and LIBS (elemental composition) can be complementary, increasing the information power [56]. This combination of techniques, called a hyphenated or tandem approach, has been already successfully used for the analysis of minerals [57] and cultural heritage objects [58]. Hoehse *et al.* [59] constructed a LIBS-Raman system with a two-arm Echelle spectrometer equipped with single CCD camera. Pořízka *et al.* [60] used laser-based techniques (LIBS and Raman spectroscopy) in tandem for obtaining both elemental and molecular information of the algal strain *Trachydiscus minutus.* suspended in water. Raman spectroscopy can be used to obtain the molecular composition of the sample under study, e.g., information on the lipid content inside algal cells. The elemental composition can be observed employing LIBS or LA-ICP-MS. As was mentioned above, LIBS is an emerging technique for elemental analysis, with its main advantage being the possibility of fast *in-situ* measurement. It has to be noted that LA-ICP-MS or LA-ICP coupled to Optical Emission Spectrometry (LA-ICP-OES) can be advantageously used also to validate the LIBS outcomes, mainly in the first stages of the research and development of new LIBS applications. The classification of the algal strains based on their spectra, *i.e.*, spectrochemical fingerprint in the sense of elemental or molecular composition, can be also provided by employing standard chemometric algorithms, such as principal component analysis (PCA) [61] and partial least squares (PLS) algorithms [61,62]. Chemometrics and their applications are further discussed in the text.

In this review the literature was surveyed for recent developments and results in research utilizing selected laser-based techniques (mainly LIBS and Raman spectroscopy, partly LA-ICP-MS) for analysis of the algal biomass or calcified coralline algae. ICP-OES was used as a supervising technique for analyzing LIBS results with chemometric.

Using LIBS and Raman spectroscopy techniques, one is able to monitor *in-situ*, on-line and in real time the spectral evolution of the major/minor elements (LIBS) and in addition chemical composition of the sample (Raman spectroscopy). Moreover, in these approaches, knowledge about the relation between elemental/chemical composition and spatial location is achieved. This enables to measure time-course data so that monitoring changes of sample over time (which could be related to spatial position) introduced by environmental/nutritional influences. On the contrary, when other approaches are used for analysis where the part of a sample is dissolved/pelleted and consequently analyzed using for instance methods based on atomic absorption spectroscopy (AAS) or MALDI information about spatial location and time evolution is hard to obtain or even completely lost.

## 2. Laser-Induced Breakdown Spectroscopy

The spectrochemical analytical technique LIBS, which is based on generating a laser-induced plasma (LIP) by high energy laser pulses and subsequent time-resolved spectral analysis of the LIP emission, can be used to analyze materials in any state of matter [63–67]. A LIP spectrum containing atomic and ionic emission lines may provide qualitative and quantitative information about the elemental composition of the sample in real-time and *in-situ*. In recent years, LIBS technique has gained its position among other spectroscopic techniques due to its advantages; such as simple and robust instrumentation, fast and precise analysis, no need for the sample preparation, the capability of on-site application and remote/stand-off detection [49]. Due to the relative simplicity of the whole measurement process, a movable remote (or stand-off) system can be constructed and employed for the analysis of environmental samples [68,69].

The utilization of LIBS in various fields is summarized in the review articles [56,70–72]. The technique has already proved its capability for the analysis of biological samples [73,74] and in biomedical applications [75]. Review on the femtosecond (fs) LIBS (physics of the laser-induced plasma, applications and perspectives) was introduced in [76]. The utilization of fs-laser source in a LIBS measurement leads to significant suppression of the matrix effect. Though, there exist multi ways how to overcome the matrix effect occurring in ns-LIP [56], e.g., Laser-Ablation LIBS (LA-LIBS).

To the best of our knowledge, only a few pioneering works have recently been performed in the analysis of algae employing LIBS. Surveying the literature for the LIBS measurements, multiple arrangements and several approaches can be found for direct measurements of algal strains. One possibility is to dry the algal biomass to produce a thin film [60] or to press dried algal biomass into the pellets [77,78]. Garcimuno *et al.* [77] measured natural watercourse algae with added standard solutions of Cu. The analytical figure of merit, limits of detection, were obtained and claimed to be in the units of ppm. Niu *et al.* [78] produced internal standards by adding known amount of Sr into dried biomass of two different algal strains, *Chlorella* and *Sargasso*. Both strains were standardized samples obtained from National Institute for Environmental Studies (NIES) in Japan. After drying, the algal powder was pressed into pellets, and measured by LIBS. The amount of Sr in the unknown sample was then successfully evaluated using the constructed calibration curves. The approach pressing the pellets prior the LIBS analysis, however, is time-demanding and not-applicable for *in-situ* and time-course analysis [77,78]. The most straightforward way is to measure the algae directly, in the water suspension. Pořízka *et al.* [60] observed the elemental composition of algal strain *Trachydiscus minutus* (Bourrelly) measured with LIBS in three ways, (i) the algal sample was dried and deposited into the thin biofilm; (ii) the suspended algae was measured in the liquid jet; and (iii) in bulk (where laser-induced plasma was produced on the surface of the liquid). Elements of biological significance (Ca, K, Mg, and Na) were determined as well as the trace amounts of potentially toxic metal (Cu). The results of LIBS analysis of algal strains are listed in Table 1.

One consideration that needs attention when using LIBS for quantitative analysis are “matrix-effects”, and these have been the subject of much discussion in all branches of spectrochemical analysis. These are the effects on the spectra associated with the combined physical and chemical properties of the target which result in different dynamics of laser/matter interaction and consequent ablated mass values, plasma formation, and its properties. They cause outliers in calibration plots if the samples are not chosen with similar composition. Extreme cases are seen when calibration plots for the same element are obtained from totally different materials. To get around these problems the composition of the calibration samples should closely match, in the sense of matrix elements content, that of the material to be analysed. Otherwise a different analytical approach has to be followed.

The composition of the sample, mainly in the sense of matrix or macro elements, is crucial in the laser/matter interaction and consecutive LIP formation and emission. The quantitative analysis of trace elements content in the algal samples is limited due to the significant influence of the matrix effect on the intensity of trace element lines. The same amount of an analyte in samples with various matrices may result in the significantly different intensity of corresponding spectral line. Then general calibration of the system for various matrices is therefore not possible. For this reason, the ways of compensating or even avoiding the matrix effect should be considered. The performance of LIBS in quantitative analysis may be improved by multivariate algorithms [64]. Though, multivariate algorithms, such as principal component regression (PCR) and partial least squares regression (PLSR), may compensate the matrix effect only to a certain extent. However, when the classification prior to the quantitative analysis is considered the main benefit may be to discriminate the samples based on their matrix elements content. Then the matrix effect may be suppressed while the calibration curve is constructed only for particular group, *i.e.*, samples with limited range of variation in the composition of matrix elements.

LIBS analysis of liquid samples is very challenging for the essential problem arising from the laser/liquid interaction. Improvement in the LIBS instrumentation for the measurements of samples in the form of liquid solutions and suspensions should lead to the improvement in the sensitivity and repeatability of the technique. Moreover, the density of the liquid suspension has to be taken into account when different ratios (water to algae) affects also the matrix. The measurement can be performed in the matrix assisted mode, as was presented in [79], where algae were deposited on the surface which matrix is considered to be supreme, or simply measured in the form of dried biofilm [60]. With this approach the promising improvement in the sensitivity and repeatability of the LIBS setup is expected. This approach was adapted in our recent measurements [60] where alga *Trachydiscus minutus* (Bourrelly) was deposited on a microscope slide and dried. Thin film of algal biomass was then analyzed utilizing a double pulse LIBS (DP LIBS) technique. Elements of biological significance (Ca, K, Mg, and Na) were detected with the highest signal for Ca (II) doublet (393.4 and 396.9 nm). Furthermore, DP LIBS was utilized for higher sensitivity and lower amount of ablated mass. Moreover, table-top LIBS setup offers in general satisfying repeatability and reproducibility of the measurement with limits of detection under the ppm level.

The ratio of algae to water content may be controlled if the algal strains are prepared under the laboratory conditions. Further problems arise when the algal strains are collected from their natural environment, which affects the elemental composition of an algal strain. Though, influence of environmental parameters could be helpful when the classification of the algal strains is of an issue, *i.e.*, in the provenance study. Then, an algal strain may be classified when the composition of matrix elements (Ca, K, Mg, and Na) is affected by the surrounding environment.

Despite the increasing popularity of LIBS within the spectroscopic groups dealing with various application fields, the use of LIBS for the analysis of algal biomass remains still unexplored. Published papers on the analysis of algae samples employing LIBS, however, proved the capabilities of this analytical technique. Both reproducibility and good sensitivity were reached when algal pellets were measured with additional trace amounts of toxic heavy metals. The approach for measuring algae in liquid suspension further strengthened the position of LIBS for *in-situ* measurement of biological samples—from a liquid jet the instrument (see further in the text) can directly measure *in-situ*, in real-time and on-line changes in algal elemental composition within the bioreactor. LIBS is a promising technique for analyzing the algae elemental composition of samples *in-situ* and in real-time, in their natural habitat. It can be useful for monitoring the cultivation process of algae and for evaluating environmental pollution.

It should be noted that in different fields, e.g., in biofuel production, the elemental analysis of algal samples is not the primary objective with respect to the molecular analysis. As detailed in Section 2.2, LIBS, with certain limitations, is capable also of direct molecular analysis. Utilizing LIBS solely for elemental analysis, however, has applications in the analysis of drinking water, evaluation of the waste waters and its treatment control, the so-called bioremediation, trace elements detection in marine algae, *etc.*

### 2.1. Laser-Induced Breakdown Spectroscopy of Liquid Samples

An effective LIBS arrangement using a vertical thin liquid flow for measurement of trace amounts in water solutions has already been used [60,80]. Thus, LIBS has proved its applicability for detection of trace elements in aqueous samples, but for the rapid *in-situ* and real-time analysis of biological samples suspended in water, *i.e.*, algae, it is necessary to further develop LIBS systems, so that they are capable of measuring samples in the liquid state of matter.

Despite the general problems with plasma generation in liquid samples, the capability of LIBS for analysis of liquid samples has been tested and improved for more than thirty years. A detailed literature survey on the analysis of liquids is beyond the scope of this review, however, more comprehensive review on the analysis of liquid can be found in [56]. Practically, three different approaches of liquid sample measurements can be employed: (1) creation of the plasma in a bulk of the liquid; (2) on the steady surface of the liquid; or (3) on the surface of a thin laminar jet.

Both approaches, in bulk and on surface analysis, suffered from the sedimentation process when the suspended specimen in the liquid settled down to the bottom, making the sample inhomogeneous and the measurement irreproducible. This fact led to the introduction of the thin laminar vertical flow of a liquid [81–85]. Limits of detection found in selected articles are in the tenths or units of ppm for heavy metals (Pb, Cu, and Cr) and elements of biological significance, matrix elements of algae, (Ca, Na, Mg, and K). Preliminary LIBS measurements of algae suspended in liquid suspension utilizing the liquid jet and steady surface approaches were reported [60]. In this article, the peaks of H_α_, H_β_ and O lines were obtained with highest intensity.

### 2.2. Laser-Induced Breakdown Spectroscopy for Molecular Analysis

The analysis of a LIP emission provides information on the elemental composition of the sample. Molecular bands can be as well detected in a LIP radiation [56]. The molecular structural information of a sample is broken after the impact of a laser pulse. The newly formed LIP is composed of ions, atoms, and electrons. As the LIP cools down, ions recombine and molecules may be formed further in LIP temporal evolution. Consequently, the radiation corresponding to excitation states of molecular bands is detected. The molecular bands occur in later stages of the LIP formation. Debras-Guedón and Liodec [86] made series of measurements of molecular radiation, CN and AlO. However, CN bands were present in the plasma of carbon samples. Different timing of the LIBS experiment should be utilized for molecular analysis compared to conventional elemental analysis. Elements originating from the ambient gas surrounding the sample are as well ablated to create a consequent LIP. Those elements then react with the elements from the sample and form molecular bonds. Then, detected molecular radiation does not directly reflect the molecular structural information of the sample. Therefore, significant uncertainty may be introduced to the computation. Molecular analysis is not so frequent in LIBS applications, though this kind of analysis has already been tested. It was already proved that the CN band is a consequence of recombination among C_2_ in the plasma, *i.e.*, ablated C from the sample, and N_2_ originating from the ambient air [87]. Moreover, Baudelet *et al.* [88] in their work suggest the CN band as a reliable marker for the observation of biological samples.

Doucet *et al.* [89] coupled LIBS measurement with chemometrics (principal component regression and partial least squares regression) in order to obtain more reliable quantitative molecular prediction. In this work they analyzed 18 standardized pharmaceutical samples with emphasis on CN band emission. Kongbonga *et al.* [90] analyzed different types of oils and saccharose dissolved in water with an emphasis on direct detection CN (in the region 388 nm) and C_2_ (516.6 nm) bonds. The attempt to correlate the intensity of molecular band with the amount of fatty acid in the sample was done. However, no calibration curve was given due to a low range in the amounts of fatty acids. The detection of CN and C_2_ bonds together with the theory of the chemical processes involved in forming those molecular bands in LIP were presented by several authors [91–95]. In the wider context, the detection of molecular bonds (e.g., C_2_) utilizing LIBS could be correlated under well specified circumstances to the real amount of fatty acids.

Utilization of laser-based methods, LIBS and Raman spectroscopy, for obtaining complete chemical information of algae has already been published [60]. In this work, the analysis of molecular information was delivered by Raman spectroscopy. Utilization of Raman spectroscopy with LIBS beneficially in tandem was already reviewed by Hahn and Omenetto [56] for various applications (such as archeology, cultural heritage, mineralogy and soil analysis). Nevertheless, based on the proposed theory, LIBS can—under well specified circumstances—provide both elemental and molecular information. There are several molecular bands (CN, C_2_, CO, CO_2_) which can be detected in the LIP radiation. The concentration of molecules within a LIP could be obtained when the LIBS measurement is supervised with Raman spectroscopy or GC-MS. Advanced statistical algorithms, such as PLS and PCR (principal components regression), could be used for that purpose.

Concluding the LIBS section, LIBS instrument could be used in bioremediation and environmental pollution monitoring. The sensitivity of the technique is satisfactory with the detection limits in the units of ppm. However, a robust LIBS setup with good reproducibility has to be constructed. As it was concluded by Hahn and Omenetto [56], the LIBS device is capable of quantitative analysis, however it is considered to be the only vulnerable feature of LIBS, therefore further research should be concentrated in this direction. Moreover, the matrix effect is of an issue when the quantitative analysis of the trace element is needed [56,64]. Nevertheless, it is possible to avoid or compensate the matrix effect to a certain extent in many ways, e.g., LA-LIBS, fs-LIBS, matrix assisted LIBS, and chemometric algorithms. Despite its limitations, LIBS is capable of direct and fast *in-situ* analysis without any need of sample preparation. Moreover, it is possible to classify various samples based on their chemical fingerprint provided by LIBS.

The performance of a LIBS device for *in-situ* analysis should be adapted to a case study rather than to a general use. Then, LIBS should be in the first stage of the research supervised with another technique (such as ICP-OES, GC-MS, *etc.*) to obtain the reference results and then to construct the supervised library of algal strains. Consequently, LIBS instrument can provide reliable real-time analysis.

## 3. Laser Ablation Inductively Coupled Plasma Based Techniques

To quantify the total content of elements in algal samples, numerous analytical techniques have recently been employed. Most of them, such as solution analysis by ICP-OES and ICP-MS, require sample decomposition and dissolution. The main drawback is the relatively demanding and laborious sample pre-treatment. After rinsing in ultra-pure water to remove salts and oven-drying, the samples are homogenized, grinded and then weighed [96,97]. Some samples are extracted [98]. The next step is the decomposition procedure, usually the acid digestion [99] or microwave assisted acid digestion [98]. Other treatments such as the slurry sampling technique, acid leaching or the enzymatic hydrolysis can be used [100]. These procedures lead to a total dissolution of the biological materials. Due to the complete decomposition of biological materials, however, the spatially-resolved analysis of elements cannot be carried out.

The solution ICP-OES technique was used to determine major (such as Ca, K, Mg) and trace elements (such as Zn, Cr, Co) in edible algae [97–100]. Using ICP-OES Michalak *et al.* [100] observed the differences of concentrations of elements of marine edible algae during the annual period in different parts of Baltic Sea. Perez *et al.* [21] utilized ICP-OES of algae from three different stations from Patagonia (Argentina) to detect the degree of contamination caused by human activities and to study the seasonal differences between Cd and Pb. ICP-OES is distinguished by ability of multi-elemental analysis of biological (algal) samples with relatively high-sensitivity and rapidness over wide concentrations ranges [98]. Better sensitivity can be acquired by solution ICP-MS. It is a very sensitive and precise analytical technique that allows simultaneous determination of trace and ultra-trace elements in algae with detection limits in the order of ng·g^−1^ [101]. Van Netten *et al.* [102] utilized ICP-MS to control heavy metals and radioactive isotopes in edible marine algae. The amounts of heavy metals in edible algae have to be controlled because of algal high affinity to heavy metals. Rodenas de la Rocha *et al.* [96] have also employed ICP-MS to analyze different elements in edible algae.

The analysis of biological samples without laborious decomposition can be performed using LA-ICP-MS/OES. These analytical techniques are widely used for a trace elemental analysis of solid samples with high spatial resolution (typically below 20 μm [50]). Moreover, LA-ICP-MS allows also a multi-element analysis of biological samples with no or little sample preparation and enables rapid analysis in real time with high spatial resolution. This advantage enables the observation of the evolution of appropriate biological samples (e.g., calcified algae) during their lifetime, which aids in the understanding their life cycle (series) or living conditions.

LA-ICP-MS was used on coralline red algae to detect climatic condition changes. Coralline red algae represent an ideal organism that occurs in mid- to high-latitude oceans. Their asset is their longevity and their incremental growth pattern. They are widely distributed in the coastal regions worldwide. Part of their skeleton is constituted of high content Mg-calcite. This skeleton grows over their lifespan. Coralline red algae do not suffer from the drawback of the ontogenetic growth trend [103]. Usually, to detect climatic condition changes, the most plentiful species of *Clathromorphum* from North Pacific Ocean are used; they can grow up to 850 years (based on radiometric dating). They can record climate information during an annual period [50–54].

The ratio of Mg/Ca is used to record temperature variation in different marine organisms [97,102]. For the detection of the sea-surface temperature Hetzinger [55] utilized coralline red alga, *Clathromorphum nereostratum*, which archives the environmental information of seawater with a high temporal resolution during its growth. Gamboa *et al.* [53] compared by LA-ICP-MS the Mg/Ca ratio in coralline red algae *Clathromorphum compactum* from two sites within the same region and showed that algae can be used as a recorders of past temperature variability. Halfar *et al.* [51] utilized *Clathromorphum compactum* to employ growth increment widths as a temperature proxy by LA-ICP-MS. They investigated the relationship between the growth and the environmental parameters. Averaged results of multiple growth increments show strong correlations with annual sea surface temperature. They showed that the highest growth rates are observed during the summer months when the sea-surface temperatures and the light intensities are the highest.

Chan *et al.* [52] and Hetzinger *et al.* [54] used LA-ICP-MS to study the Ba/Ca ratio variations in *Clathromorphum nereostratum*, and investigated temporal salinity changes. Gamboa *et al.* [53] utilized LA-ICP-MS to determine Mg/Ca ratios of *Clathromorphum compactum* to understand the North Atlantic Oscillation. Hetzinger *et al.* [50] investigated algal species *Clathromorphum compactum* and *Clathromorphum nereostratum* by LA-ICP-MS in order to compare the ratios of Mg/Ca, Sr/Ca, U/Ca and Ba/Ca. The temperature dependence of Sr/Ca and Mg/Ca ratios was evidenced. The results show that the Sr content into algal calcite is dependent on the seawater temperature. The relationship between Mg/Ca, U/Ca and Ba/Ca ratios and the sea surface temperature was not proved.

The reported limits of LA-ICP-MS detection are under the ppm range, summarized in Table 2. For the analysis of algal samples, *i.e.*, measuring the concentrations of ^24^Mg, ^43^Ca, and ^137^Ba, an Agillent 7500ce Quadrupole ICP-MS coupled with a New Wave Research UP 213 laser ablation system (213 nm, ND:YAG laser) was used [51–53]. The carrier gas was helium and the utilized laser energy density was 6 J/cm^2^. The scan speed was 10 μm/s, the spot size was 65 μm, and the pulse rate was 10 Hz. NIST SRM 610 (U.S. National Institute of Standard and Technology Standard Reference Material) glass reference material was utilized as an external standard [51,52].

LA-ICP-MS is a prospective method to study solid algal samples with high resolution and relatively low limits of detection. This method seems to be more suitable to detect coralline algae than LIBS because LA-ICP-MS has in general higher spatial resolution and can detect isotopes ratio. This is important in the study of coralline algae, because they are optimal organisms to archive and detect climatic conditions of the environment. On the other hand, LA-ICP-MS cannot be used to study algal suspensions, and needs reference methods to measure the total concentration of elements in the samples, or standard reference materials to quantify elemental concentration in the samples.

## 4. Raman Spectroscopy

Raman spectroscopy (alternatively Raman tweezers–a combination of Raman microspectroscopy with optical trapping) is a powerful and robust technique for analyzing biochemical information and revealing the molecular composition of samples under study [42,104–113].

**Figure 1.**
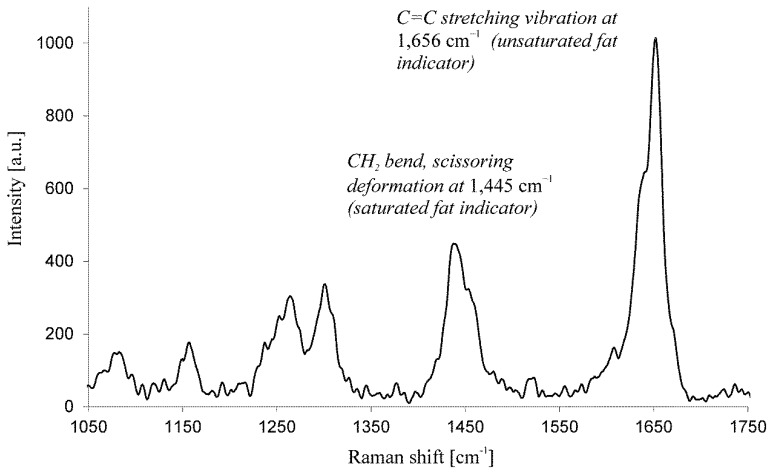
Raman spectrum of a lipid body within the algae (*Trachydiscus minutus*). From the ratio of intensities I_1656_/I_1445_ the iodine value (IV) can be estimated [42]. Here algal lipid content is close to IV ∼ 230.

Raman spectroscopy is based on the phenomenon of Raman scattering of monochromatic light (laser), which is the inelastic scattering of a photon. When there is monochromatic light incident on a target there are several possibilities for the incoming photons, if they have sufficient energy the molecules of the target can be raised to an excited electronic state and the photons absorbed, they can pass through the target without interacting or they can undergo elastic or inelastic scattering. Elastic scattering is the type of scattering that occurs most often when light is incident upon a target. In Raman spectroscopy however, inelastic scattering is exploited. In this case the molecule is excited to a virtual energy state, however this time when it relaxes it returns to a different vibrational energy state than the one that it started from. Therefore the energy of the photon emitted during the relaxation is different to the energy of the photon that caused the excitation in the first place. The scattered photon consequently has a different frequency than the excitation source and this is what produces the Raman spectrum, a plot of the frequency shift between incident and scattered light (see Figure 1).

Review articles on biological applications [114–118] and especially on algae [119] have been presented. The primary goal of this literature research is the utilization of Raman spectroscopy for obtaining the information about the amount of lipid content within the algal cell. Efficient production of algal strains with higher lipid yield could lead to lowering the prices of biofuels [3]. Therefore, techniques allowing for rapid characterization/identification of algae species are required, and specifically to determine the degree of unsaturation of constituent fatty acids in algal lipid bodies. Note that the third generation biofuels technology is based on algae that contain high oil content. Also, concerning the modern fish industry, most fish consumed inland come from fish farms. In this aquaculture industry, as the number of fish farms grow (mainly control farmed salmon products), it becomes important to guarantee that the high content of precious omega-3 fatty acids find their way into fish oils. This therefore dictates some fish dietary requirements for dedicated aqua-cultural environment. Consequently, aquafarmers feed fish, soy, and chicken oil to fish, all of which could be eliminated using algal oil. This highlights algae as a potential source from which desired omega-3 fatty acids can be extracted [120].

In 1983, Brahma *et al.* [121] reported on the measurement of the marine algae phytoplankton, employing Raman spectroscopy. Algae were measured directly in the suspension and the emphasis was given to the observation of the carotenoid pigments and chlorophyll peaks. The application of Raman spectroscopy to the analysis of photo-synthetizing organisms, such as algae, is challenging due to the underlying strong fluorescence of omnipresent pigments that might obscure the characteristic Raman spectral features. Therefore, the use of Raman spectroscopy has been limited to relatively few algal species. Accordingly, the number of published papers is relatively small, but tends to increase in recent years. Because of being in an early stage of development, these publications on Raman spectroscopy of algae are scattered over a wide range of journals, for instance [42,122–127], with the majority of work published in the last five years.

Algal strains, which could be promising candidates for biofuel production, have been so far investigated by five groups worldwide (see Table 3). The most widely studied species is *Botryococcus braunii*. The species with the highest iodine value (IV) was found to be *Trachydiscus minutus*. Thus far only two groups have been involved in systematic research on estimating the unsaturation degree/IV within algal samples (see Table 3).

The pioneering work of Heraud *et al.* [122,126], performed in 2007 in Beardall's laboratories at Monash University (Australia), was focused namely on *in vivo* Raman spectroscopy to predict the nutrient status of individual algal cells. They found that the Raman spectra of cells revealed a range of Raman bands mainly attributed to chlorophyll and carotene when 780 nm laser beam was used for excitation.

Preliminary feasibility studies on using Raman spectroscopy of algae were reported by Huang *et al.* [123]. They performed the study on two algal species, namely *Chlorella sorokiniana* and *Neochloris oleoabundans*, which could be seen as potential candidates for the biofuel production. Nitrogen-starved *C. sorokiniana* and *N. oleoabundans* samples were measured and Raman signals due to storage lipid (specifically triglycerides) were detected. The fluorescence background interrupted by sudden high-intensity fluorescence events was observed in the Raman signals from the algae. The fluorescence was acquired as a consequence of photo-bleaching of cell pigments due to prolonged intense laser light exposure; but the occurrence of the sudden high-intensity fluorescence bursts eluded full understanding.

Weiss *et al.* [124] reported Raman spectroscopy on *Botryococcus braunii* algae. In this study, authors were focused mainly on mapping the presence and location of methylated *Botryococcenes* within the colony. Specific Raman spectroscopic characteristics for *Botryococcenes* of *Botryococcus braunii* have been identified. *In vivo* lipid profiling of oil-producing algae has been described, using single-cell laser-trapping Raman spectroscopy [125].

Finally, Samek *et al.* [42] have recently demonstrated spatially resolved Raman spectroscopy to determine the effective IV in lipid storage bodies of individual algal cells. The Raman spectra were collected from different algal species immobilized in agarose gel, thus preventing them from moving out from the tightly focused region of the probe laser beam in order to maintain high spatial resolution within lipid bodies. The principal parameter characterizing the algal lipid is the degree of unsaturation of the constituent fatty acids and can be quantified by the IV. Crucially, the IV is conveniently estimated from information contained within the Raman spectra, with no need to add any chemicals to the cells. They used the characteristic peaks in the Raman spectra at 1656 cm^−1^ (*cis* C=C stretching mode) and 1445 cm^−1^ (CH_2_ scissoring mode) as the markers defining the ratio of unsaturated-to-saturated carbon-carbon bonds of the fatty acids in the algal lipids (Figure 1). For the quantitative IV determination a calibration curve was generated based on pure fatty acids of known IV, when the IV differed significantly for the various algal species. These estimates based on Raman spectroscopy were validated using the established technique of gas chromatography mass spectroscopy (GC-MS); indeed, excellent agreement was found.

As was mentioned above, the technique of Raman spectroscopy could be an excellent candidate to follow the food chain in the aquaculture industry, enabling one to monitor the IV within the food chain. Similarly, the same procedure of IV determination can be applied to monitor algae samples for biofuel production, where IV must be kept below a given limit. Moreover, it has been demonstrated [42] that various algal oils exhibit significantly different IV, which may have important implications for the food/pharmaceutical industry in obtaining 3-omega fatty acids. The main advantage of oils obtained from algae is that they are not contaminated by industrial toxins/antibiotics as some oils obtained from fish possibly could be due to the contaminated environment (industrial farmed fish or wild-caught fish).

## 5. Chemometrics for the Recognition of Algal Strains

The discussed spectroscopic techniques (LIBS, LA-ICP-MS, and Raman spectroscopy) are able to analyze extensive samples set, where each sample is represented by complex spectral information. Chemometric algorithms are used in many fields including spectroscopy, for data mining and pattern recognition within the bulky data sets [61]. In general, chemometrics are used for both qualitative and quantitative analysis.

Classification of the algal strain has already been performed. Zbikowski *et al.* [128] applied the factor analysis for the discrimination of algal strain collected in coastal and lagoon waters, based on their appearances in flame atomic absorption spectroscopy spectra (FAAS). The algal strains were collected in the Gulf of Gdansk in a short time period (2000–2003). Algal samples were then dried and digested with HNO_3_ acid. The contents of four macroelements (Ca, Mg, Na and K) and six heavy metals (Cd, Cu, Ni, Pb, Zn and Mn) were determined and used for further statistical analysis. The correlation between the concentrations of Cu, Pb and Zn in green algae and the sampling sites was observed. Heraud *et al.* [126,129] utilized Raman spectroscopy and Fourier transform infrared spectroscopy (FTIR), respectively. Chemometrics were then applied on the measured data set for accurately predicting the nutrient status of an independent individual algal strain. PCA was successfully applied as well on the near-infrared (NIR) and FTIR spectra [130]. Laurens and Wolfrum [130] used the NIR and FTIR spectra of biomass from four species to predict accurately the levels of exogenously added lipids. Salomonsen *et al.* [131] presented an extensive comparative study of alginate, a salt of alginic acid distributed widely in the cell walls of brown algae, using IR, Raman spectroscopy, NIR and NMR techniques. Chemometric algorithms, partial least squares discriminant analysis (PLS-DA) and PCA were then used to accurately predict the nutrient status of the cells from the Raman spectral data.

Concluding, chemometric algorithms may be of help in handling bulky data sets and revealing latent variables and relations among the biological samples. As stated above, LIBS is capable of providing information about the overall elemental composition of the sample, the so-called chemical fingerprint. The composition of matrix elements Ca, K, Mg, and Na could differ according to the particular measured algal strain [60]. The classification of algal strains based on their spectra is possible while employing the standard chemometric algorithm, such as principal component analysis. To the best of our knowledge, chemometric algorithms have not been used so far for the analysis and discrimination of algal strain based on their LIBS measurements. Nevertheless, successful utilization of LIBS for classification of biological samples has already been published [88,132–135].

### 5.1. Discrimination of Four Algal Strains by LIBS

**Figure 2.**
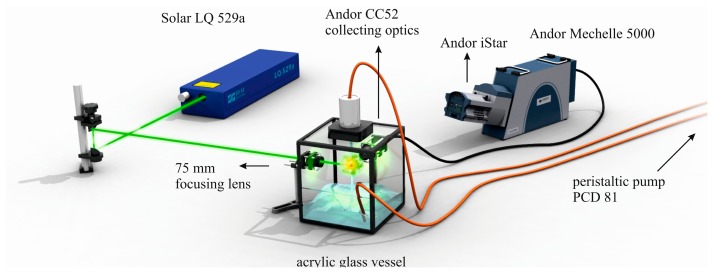
Schematic diagram of the so called liquid LIBS system [60].

**Figure 3.**
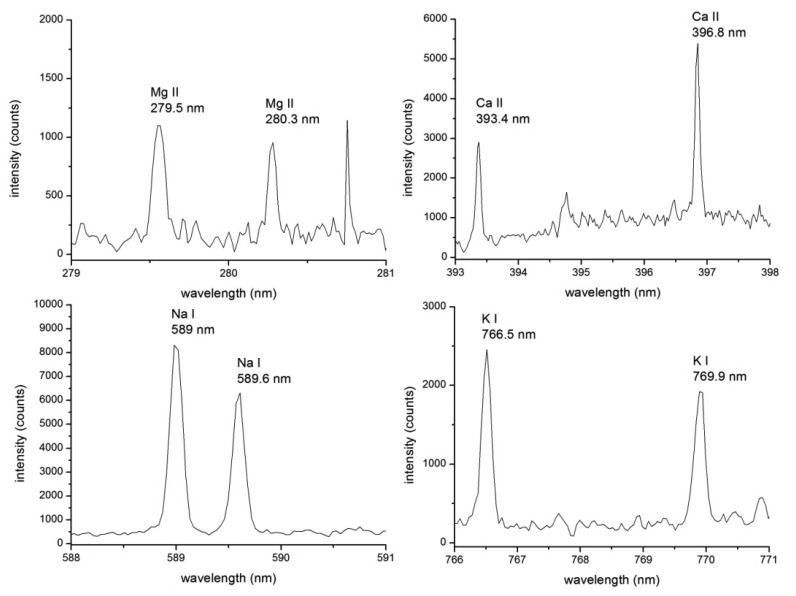
Typical spectrum of algae with the emphasis given to the matrix elements (Mg, Ca, Na, and K).

Description of employed LIBS system, Figure 2, and related preparation of the four algal strains has already been published [60]. Four algal strains (*Chlarydomonas reinhardti (ChR)*, *Chlorococuum zurek (ChZ)*, *Desmodesmus quadratic (DQ)*, *Haematococcus pluralis (HP)*) were prepared under the same laboratory conditions. The samples were measured in the form of liquid suspensions with LIBS device where thin liquid jet was utilized. In this experiment, ns-laser pulse (Solar LQ 529a; operated at 532 nm, 10 ns, 50 mJ, ∼65 GW/cm^2^) was focused with 75 mm planoconvex lens into a tight spot (100 μm). Radiation of a LIP was collected by using a large aperture collector-collimator (Andor CC52, F/2). Collected light was then spectrally resolved on the echelle grating (Andor Mechelle 5000; F/7, λ/Δλ = 6000) and detected by an ICCD (Andor iStar 734). The temporal gating of the LIBS experiment was as follows: the gate delay of 3 μs and the gate width of 10 μs. Each measurement consists of 50 spectra in an accumulation while each measurement was 20 times repeated to obtain robust statistical dataset. A typical spectrum of an algal strain is depicted in Figure 3, where matrix elements (Ca, K, Mg, and Na) are highlighted. During the data processing, the spectra were normalized to their integral intensities and averaged, and then four spectra per sample were obtained. Lines of matrix elements, listed in Table 4, were fitted with pseudo-Voigt profile and their intensities were calculated as the area under the peak with the background subtraction using custom MATLAB (version R2012a) software. Four spectra per each sample were organized as rows in the data matrix and its columns refer to individual variables. The range of each variable was normalized to unity and then mean-centered. The data matrix was analyzed with PCA to reveal possible latent variables among the data and to provide the discrimination of the samples (for detailed description of this procedure see further paragraph). This analysis was done employing MATLAB software customized with Self-Organizing MAP (SOM) toolbox [136] (Helsinki University of Technology, Finland) for multivariate analysis.

Working with the whole data set can result in a PC space where various samples may be assigned to one group. In other words, the least squares property of PCA algorithm highlights the most significant variation among the data. For this reason, the less significant variation is overshadowed, *i.e.*, has lower impact on the classification in a newly constructed PC space. To overcome this problem one can utilize the approach suggested by Multari *et al.* [132] and used as well in related work by Ollila *et al.* [137]. There, any cluster is removed from the computation when it is successfully assigned to a distinct group in respect to the rest of the data set. Then the PCA algorithm is applied again on the reduced dataset. This leads to simplification of the variation in the dataset. In other words, the variation responsible for the distinct separation of a group withdrawn from the computation is not present anymore. This results in the increase of the significance of a formerly less significant variation among the rest of the data. This process is repeated until all of the samples are successfully classified. By employing this algorithm for data classification we can proceed further in our investigation.

**Figure 4.**
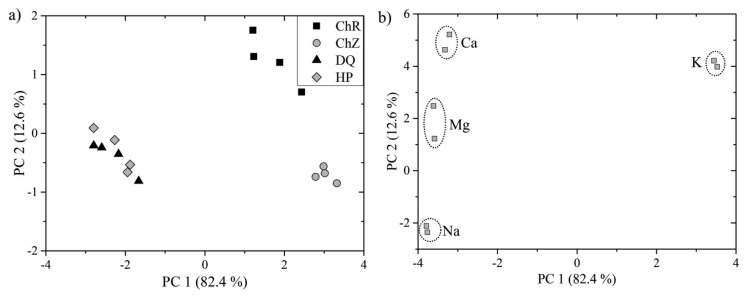
PCA of four algal strains based on their LIBS measurement: (**a**) scores; (**b**) loadings.

PCA was then applied on the data matrix constructed from the LIBS data. Two distinct groups are clearly visible when investigating resulted PCA scores in Figure 4a. The first two principal components describe 95% of overall variation among the data. This suggests that the discrimination of algal strains into three distinct groups is possible. However, data points representing algal strains *DQ* and *HP* are strongly overlapping. This may be a consequence of the similarity in the matrix elements and moderate repeatability of the LIBS measurement.

PCA analysis of LIBS spectra, *i.e.*, the clustering of the LIBS measurements, was then emulated by the PCA analysis of the ICP-OES measurement (not shown in this article). As in the case of LIBS measurement, the main emphasis was given to the signal intensities of the matrix element lines (namely Ca, K, Mg, and Na lines). The scores plot indicated that the composition of *DQ* and *HP* is more similar in the sense of matrix elements, *i.e.*, the data points are closer to each other, than the composition of *ChZ* and *ChR*, whose data points are distinctly separated in the newly created PC space. Those results coincided with the results of PCA applied to the LIBS data, where LIBS measurements of *ChR* and *ChZ* are distinctly separated compared to the overlapping LIBS data of *DQ* and *HP.* Therefore, the repeatability of the LIBS measurement under given conditions still could be improved for reliable classification using PCA.

## 6. Conclusions and Future Prospects

Algae are considered to be promising alternative sources to corn and soybean for the next generation biofuel production. Third generation technology may be based on algal biomass, which is rich in polyunsaturated fatty acids. The algal biomass can be grown without competing for arable land (e.g., industrial waste waters can be used for the cultivation instead of the land suitable for growing food crops). Also, algae have the potential to decontaminate polluted water, because their cellular wall exhibits high affinity to metal cations. They are also widely used as a dietary supplement and in the drug industry. Moreover, calcified coralline algae can be utilized to detect climatic condition changes due to their longevity.

A comprehensive review on the analysis of algal biomass was given, preferably for the application in the fields of biofuels and bioremediation. The main aim of this review is focused on laser-based techniques for elemental and molecular analysis. It was shown that further development of methods for monitoring the elemental/chemical composition of the algal biomass is necessary. Each individual algal strain has different properties and reacts differently with its environment. Current laser-based spectroscopy techniques presented here such as LIBS, Raman spectroscopy and LA-ICP-OES/MS represent powerful tools for fast and complete analysis of biological samples and with certain limitations, can be adopted for effective analysis of algae biomass.

LIBS technique provides information primarily about the elemental composition. However limited information about the molecular structure can be also obtained. LIBS can serve as a robust, remote and rapid method for *in-situ*, on-line and real-time elemental analysis. Furthermore, portable LIBS equipment, employing a water jet, can be constructed for the fast elemental analysis or the algal identification in the field, mainly for the bioremediation application. Note that in the primary stage of the LIBS development LA-ICP-MS/OES can be also advantageously used to validate the LIBS outcomes. When the supervised spectral libraries are created, LIBS can stand alone as a robust, remote and rapid device for *in-situ* elemental analysis.

Raman spectroscopy (alternatively Raman tweezers – a combination of Raman microspectroscopy with optical trapping) is suitable for *in vivo* analysis of algae molecular composition in a non-destructive way. Recently, the primary goal of Raman spectroscopy was the determination of the lipid content within the algal cells. Spatially resolved Raman spectroscopy utilized for the determination of the iodine value, *i.e.*, lipid storage composition in the algal bodies, has a potential importance, especially in regard to third generation biofuels technologies. Raman spectroscopy as the only from the presented methods does not influence viability of living cells and can be combined with optical tweezers to sort individual cells according to their lipid content for subsequent breeding.

Chemometrics become more popular and irreplaceable in the spectral data mining. Chemometric algorithms may be an indispensable part of a robust analysis. Discrimination of different algal strains by LIBS or Raman spectroscopy using chemometric algorithms is also provided.

Pioneering works combining some of these approaches (e.g., LIBS and Raman spectroscopy) have already been published and the results, which are discussed above, show that those approaches can open new directions of bioanalytical remote measurement of algae.

## Figures and Tables

**Table 1.** The list of articles focused on the analysis of algae employing LIBS.

Algal Strain	Ref.	Pretreatment of Algae	Matrix Elements	Minor Elements	Trace Element	LOD (ppm)
*Trachydiscus minutus* (Bourrelly)	[60]	dried biofilm. liquid jet, surface of bulk liquid	Ca, K, Na, Mg	-	Cu	-

watercourse algae strain not specified	[77]	dried and pressed to pellets	Mg, K, Na, Fe, Si	Al, Mn, Ti, V	Cu, Cr, Pb, Zn	Cu: 9 ± 2

*Chlorella*, NIES No. 3 *Sargasso*, NIES No. 9	[78]	dried and pressed to pellets	Ca	-	Sr	-

**Table 2.** Limits of detection (LOD) obtained by LA-ICP techniques taken from selected references.

Samples	Isotope	LOD [ppm]	Reference
*Clathromorphum compactum, Clathromorphum nereostratum*	^24^ Mg	0.16	[50]
	^43^ Ca	54.9	
	^88^ Sr	0.04	
	^238^ U	0.016	
	^137^ Ba	0.13	

*Clathromorphum compactum*	^24^ Mg	0.16	[51]
	^43^ Ca	54.9	

*Clathromorphum nereostratum*	^24^ Mg	0.02	[52]
	^43^ Ca	5.47	
	^137^ Ba	0.01	

*Clathromorphum compactum*	^24^ Mg	0.16	[53]
	^43^ Ca	54.9	

**Table 3.** The list of articles focused on the analysis of algae employing Raman spectroscopy.

Algae Species	Reference	Estimate of the Degree of Unsaturation/Iodine-Value of Algal Oil
*Dunaliella tertiolecta*	[122,126]	No

*Chlorella sorokiniana Neochloris oleoabundans*	[123]	No

*Botryococcus braunii*	[124]	No

*Botryococcus braunii Neochloris oleoabundans Chlamydomonas reinhardtii*	[125]	Yes

*Trachydiscus minutus Botryococcus sudeticus Chlamydomonas sp.*	[42]	Yes

**Table 4.** Table of matrix elements utilized in multivariate analysis.

Element	Wavelength (nm)
Mg (II)	279.5
Mg (II)	280.3
Ca (II)	393.4
Ca (II)	396.8
Na (I)	589
Na (I)	589.6
K (I)	766.5
K (I)	769.9
